# DNA Methylation in Oocytes and Liver of Female Mice and Their Offspring: Effects of High-Fat-Diet–Induced Obesity

**DOI:** 10.1289/ehp.1307047

**Published:** 2013-12-06

**Authors:** Zhao-Jia Ge, Shi-Ming Luo, Fei Lin, Qiu-Xia Liang, Lin Huang, Yan-Chang Wei, Yi Hou, Zhi-Ming Han, Heide Schatten, Qing-Yuan Sun

**Affiliations:** 1 State Key Laboratory of Reproductive Biology, Institute of Zoology, Chinese Academy of Sciences, Beijing, People’s Republic of China; 2 University of Chinese Academy of Sciences, Beijing, People’s Republic of China; 3 Department of Veterinary Pathobiology, University of Missouri, Columbia, Missouri, USA

## Abstract

Background: Maternal obesity has adverse effects on oocyte quality, embryo development, and the health of the offspring.

Objectives: To understand the underlying mechanisms responsible for the negative effects of maternal obesity, we investigated the DNA methylation status of several imprinted genes and metabolism-related genes.

Methods: Using a high-fat-diet (HFD)-induced mouse model of obesity, we analyzed the DNA methylation of several imprinted genes and metabolism-related genes in oocytes from control and obese dams and in oocytes and liver from their offspring. Analysis was performed using combined bisulfite restriction analysis (COBRA) and bisulfite sequencing.

Results: DNA methylation of imprinted genes in oocytes was not altered in either obese dams or their offspring; however, DNA methylation of metabolism-related genes was changed. In oocytes of obese mice, the DNA methylation level of the leptin (*Lep*) promoter was significantly increased and that of the *Ppar-*α promoter was reduced. Increased methylation of *Lep* and decreased methylation of *Ppar-*α was also observed in the liver of female offspring from dams fed the high-fat diet (OHFD). mRNA expression of *Lep* and *Ppar-*α was also significantly altered in the liver of these OHFD. In OHFD oocytes, the DNA methylation level of *Ppar-*α promoter was increased.

Conclusions: Our results indicate that DNA methylation patterns of several metabolism-related genes are changed not only in oocytes of obese mice but also in oocytes and liver of their offspring. These data may contribute to the understanding of adverse effects of maternal obesity on reproduction and health of the offspring.

Citation: Ge ZJ, Luo SM, Lin F, Liang QX, Huang L, Wei YC, Hou Y, Han ZM, Schatten H, Sun QY. 2014. DNA methylation in oocytes and liver of female mice and their offspring: effects of high-fat-diet–induced obesity. Environ Health Perspect 122:159–164; http://dx.doi.org/10.1289/ehp.1307047

## Introduction

The World Health Organization has reported that obesity, defined as abnormal or excessive fat accumulation that may impair health, has nearly doubled since 1980, and nearly 300 million women were obese in 2008 (World Health Organization 2013). Several years ago obesity and overweight was a problem in developed countries, but it has now become a problem in the entire world. Obese humans are prone to type 2 diabetes, hypertension, cardiovascular disease, and other disorders or diseases (Howie et al. 2009), and these conditions can be transmitted to the future generations (Fullston et al. 2012; Howie et al. 2009).

Obesity is a well-established cause of sub-fertility in humans and animals. In mice fed a high-fat diet (HFD) for 16 weeks, ovulation rate, embryo development, placental function, ovarian function, and mitochondrial function were affected in oocytes (Cardozo et al. 2011; Igosheva et al. 2010; Jungheim et al. 2010; Minge et al. 2008). Dunn and Bale (2009) reported that offspring of obese female mice showed a significant increase in body length. In humans, similar results were reported for oocytes from mothers with a higher body mass index (BMI) (Wattanakumtornkul et al. 2003), and children of women with high BMI tended to accumulate more fat by 9 years of age than did children of women with lower BMI (Gale et al. 2007). These reports show that obesity causes female subfertility and also that these adverse effects can be inherited by the offspring.

Obesity can be caused by genetic mutations (Graff et al. 2013), but the environment and life style are also key reasons for obesity. Currently, overweight and obesity are attributed mainly to lifestyle factors such as excessive consumption of high-carbohydrate food, low physical activities, and other factors (McAllister et al. 2009). Several studies have provided evidence that macro- or micronutrients induce epigenetic changes in offspring (Heijmans et al. 2008; Tobi et al. 2009; Waterland and Jirtle 2003; Waterland et al. 2006). Therefore, epigenetic alterations may be an important link between the environment and genes by which obese parents transmit deleterious conditions to their children.

Genomic imprinting is a parental origin–specific gene-marking phenomenon that is crucial for normal mammalian development. Differentially methylated regions (DMRs) of imprinted genes are methylated on either the paternal or maternal allele (Reik et al. 2001; Sasaki and Matsui 2008). The DNA methylation status is established during gametogenesis and early embryo development (Lucifero et al. 2002). However, methylation patterns of genomic imprinting genes tend to be altered by a deleterious environment or manipulation (Anckaert et al. 2010; Khosla et al. 2001). The detailed mechanisms underlying these changes are still unknown.

On the basis of previous reports (Fullston et al. 2012; Howie et al. 2009), we hypothesized that maternal obesity may impair DNA methylation of imprinted genes in oocytes and that it can be transmitted to the offspring. To test our hypothesis, we used mice with HFD-induced obesity, a widely used animal model (Igosheva et al. 2010; Jungheim et al. 2010; Minge et al. 2008). We investigated the methylation patterns in DMRs of paternally imprinted gene *H19*, maternally imprinted genes *Peg3* (paternally expressed 3), *Snrpn* (small nuclear ribonucleoprotein N), *Igf2r* (insulin-like growth factor 2 receptor), and *Peg1* in oocytes of control and obese animals and their offspring. Because other studies have shown that the expression of leptin (*Lep*) and *Ppar-*α (peroxisome proliferator-activated receptor α) is regulated by DNA methylation in their promoters and that the two genes are correlated to metabolism (Burdge et al. 2009; Cordero et al. 2011a, 2011b), we also investigated DNA methylation of these two genes. We also investigated DNA methylation patterns of intracisternal A particle (IAP) in oocytes.

## Materials and Methods

Mice provided by the Beijing Vital River Experimental Animals Centre (Beijing, People’s Republic of China) were housed under conditions of 12 hr light and 12 hr dark in a temperature- (23 ± 1°C) and humidity- (60 ± 5%) controlled room. All procedures were reviewed and approved by the Ethics Committee of the Institute of Zoology, Chinese Academy of Sciences. Mice were treated humanely and with regard for alleviation of suffering.

*Obese mice*. Weaned female CD-1 mice, three per cage, were randomly divided into two groups and fed with either an HFD (D12492; Research Diets, New Brunswick, NJ, USA) or a control diet (CD) for 12 weeks (for composition of diets, see Supplemental Material, Table S1). We analyzed blood glucose using an Accu-CHEK Active glucometer (Roche Diagnostics, Mannheim, Germany) as described previously (Ge et al. 2013).

*Oocyte and liver collection*. Female mice were superovulated by an intraperitoneal injection of 8 IU pregnant mare serum gonadotropin followed by an injection of 8 IU human chorionic gonadotropin (both from Tianjin Animal Hormone Factory, Tianjin, China) 46–48 hr later (100 μL/mouse per injection). After 13–14 hr, mice were sacrificed by cervical dislocation and oocytes at the second metaphase of meiosis (MII) were collected from oviductal ampullae. Cumulus cells were removed using 1 mg/mL hyaluronidase (Vergara et al. 1997). Oocytes were washed in M2 medium (Sigma Chemical Company, St. Louis, MO, USA) until no cumulus cells were observed in the medium; oocytes with attached cumulus cells were discarded. Oocytes were then counted under the microscope. For each analysis, we used approximately 100 oocytes from 10 mice. When female offspring (from a separate group of mice) were 7- to 8-weeks of age, oocytes were collected as described above. For analysis, we used approximately 100 oocytes from 10 mice, representing five litters per group. Liver was collected at the same time.

*Generation of offspring*. The obese (HFD; *n* = 20) and control (*n* = 16) females, which had similar glucose levels, were mated with the same group of control male mice. The time at which the vaginal plug was observed was defined as gestational day 0.5. Pregnant mice eating the same diet (HFD or control) were housed in a single cage and continued on the same diet during gestation and lactation. Offspring were weaned at 21 days of age and housed three per cage. After weaning, both groups were fed the control diet.

*Bisulfite treatment and polymerase chain reaction (PCR) amplification*. Oocytes of HFD and CD dams and their female offspring were subjected to bisulfite treatment and PCR analysis as described previously (Ge et al. 2013). Briefly, protein K was added to tubes containing five oocytes and incubated for 40 min at 37°C. Each sample was then denatured with 3 M sodium hydroxide at 37°C for 15 min and modified by bisulfite solution [2.5 M sodium metabisulfite (Merck Millipore, Darmstadt, Germany), 125 mM hydroquinone (Sigma) at pH 5]. A total of approximately 100 oocytes (representing 10 mice/group) were used for each gene analysis.

Liver DNA from HFD offspring (OHFD) and CD offspring (OCD) was modified using the EZ DNA Methylation-Direct™ Kit (Zymo Research, Irvine, CA, USA) according to the manufacturer’s instructions. Modified DNA was then used as a template in nested-PCR amplification. Primers are listed in Supplemental Material, Table S2.

*Combined bisulfite restriction analysis (COBRA) and bisulfite sequencing.* COBRA and bisulfite sequencing were carried out as described previously (Ge et al. 2013). Briefly, we digested the PCR product using one or two endogenous restriction enzymes (*Taq*^α^I, *Rsa*I, *Bst*BI, or *Bst*UI). The PCR product was then cloned to T vector and sequenced (Invitrogen, Beijing, China). Spermatozoa were used as a control; some spermatozoa samples were digested and some were not.

*RNA purification and quantitative real-time PCR (qRT-PCR)*. RNA was extracted from livers using the DNA Tissue Kit (Tiangen Biotech, Beijing, China) according to the manufacturer’s instructions. The first cDNA strand was synthesized using Superscript II (Invitrogen). qRT-PCR was carried out using a Roche LightCycler 480 (Roche Diagnostics). Triple samples were analyzed for each gene, and we used the glyceraldehyde-3-phosphate dehydrogenase (*GAPDH*) housekeeping gene as a control. The expression level was evaluated by 2^–△△Ct^ (Ge et al. 2013). The primers are listed in Supplemental Material, Table S2.

*Statistical analysis*. Data are represented as mean ± SD. The significance between groups was compared by independent-samples *t*-test. We used the chi-square test to evaluate the significant difference in methylation density between different groups. A probability level of *p* < 0.05 was considered significant.

## Results

*Body weight and MII oocytes of obese dams and their offspring*. The average body weight of HFD dams was significantly higher than that of CD dams (see Supplemental Material, Figure S1A). After superovulation, the number of MII oocytes in obese dams was significantly lower than that in CD dams (*p* < 0.01; see Supplemental Material, Figure S1B). In the female offspring, the number of MII oocytes was similar between the two groups (see Supplemental Material, Figure S1C). However, at 12 weeks of age, the average body weight of females and males was significantly higher (26.3 ± 8.4% and 19.4 ± 5.5%, respectively; *p* = 0.084) in OHFD mice than in OC mice (see Supplemental Material, Figure S1D,E).

**Figure 1:**
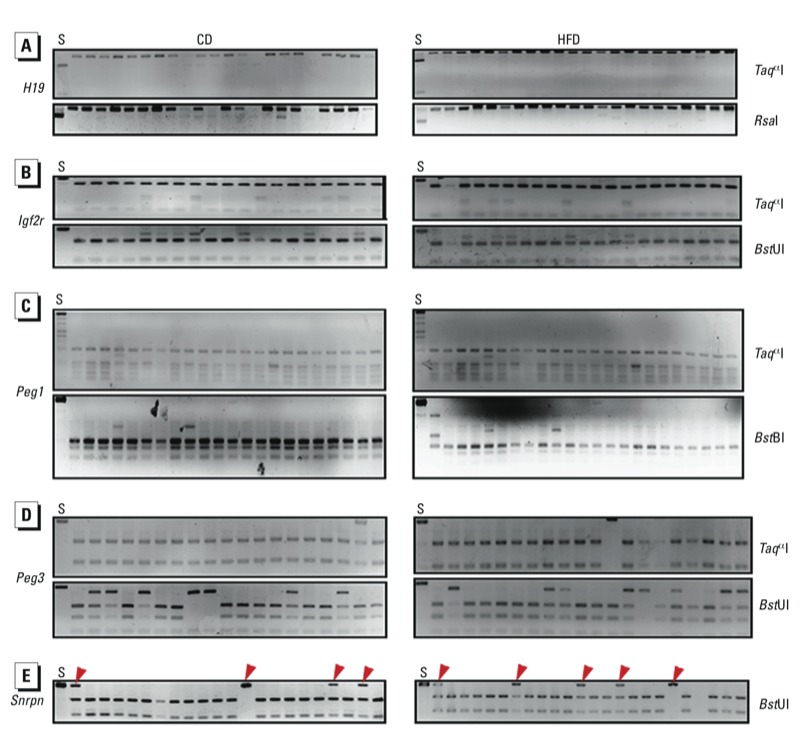
DNA methylation patterns in DMRs of paternally imprinted gene *H19* (*A*) and maternally imprinted genes *Igf2r* (*B*), *Peg1* (*C*), *Peg3* (*D*), and *Snrpn* (*E*) in oocytes from CD and HFD dams as determined by COBRA. Oocytes from 10 mice were used per analysis. Spermatozoa (*S*) were used as a control. Restriction enzymes used are shown on the right. Red arrowheads indicate undigested bands: For *H19* (*A*), the spermatozoa sample was digested and oocyte samples were undigested; for *Igf2r* (*B*), *Peg1* (*C*), *Peg3* (*D*), and *Snrpn* (*E*), the spermatozoa sample was undigested, but some oocyte samples were digested.

**Figure 2:**
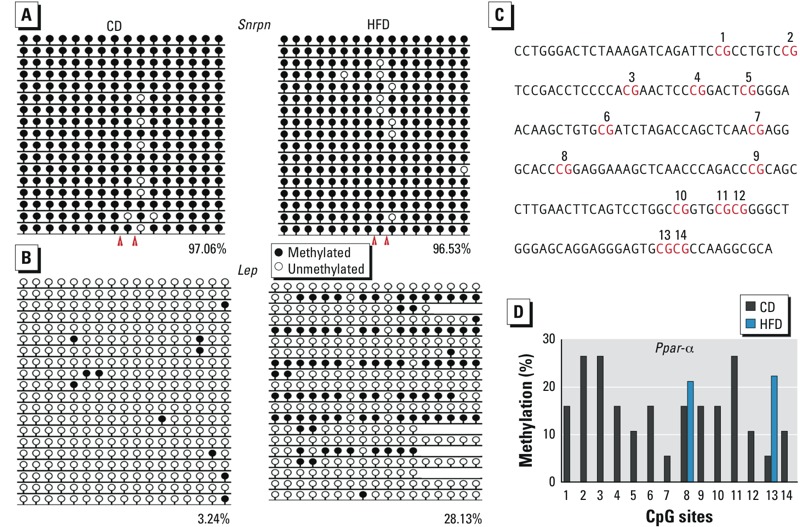
DNA methylation status of *Snrpn*, *Lep*, and *Ppar-α* in oocytes from CD and HFD dams as analyzed by bisulfite sequencing. Oocytes from 10 mice were used per analysis. (*A*) DNA methylation level of *Snrpn* (*A*; red arrowheads indicate the recognition sites of *BstUI*), and *Lep* (*B*; analyzed region located at chr6: 26009934..26010283). Numbers indicate the percentage of methylation; blank loci indicate lost CpG. (*C*) Distribution of some CpG sites in the *Ppar-α* promoter in the analyzed region; CpG sites are numbered 1–14. (*D*) Percentage of DNA methylation at the 14 CpG sites in the *Ppar-α* promoter (*C*).

*DNA methylation patterns in DMRs of imprinted genes in mouse oocytes.* For analysis of each gene, we used approximately 100 oocytes per group. For *H19* in oocytes, the bands digested by *Taq*^α^I and *Rsa*I showed that the DNA methylation in the DMR was not affected by maternal obesity (Figure 1A). Similar results were obtained in DMRs of the maternally imprinted genes *Igf2r, Peg1,* and *Peg3*, which were digested by *Taq*^α^I and *Bst*UI, *Taq*^α^I and *Bst*BI, and *Taq*^α^I and *Bst*UI, respectively (Figure 1B–D). Although some samples of oocytes from HFD dams were not completely digested by *Bst*UI for *Snrpn*, bands were similar to those observed in oocytes from CD dams (Figure 1E). Further bisulfite sequencing showed that the undigested bands of these samples were the result of DNA methylation changes at the loci of the recognition sites of *Bst*UI (Figure 2A). However, we observed no significant difference in *Snrpn* between HFD and CD oocytes (Figure 2A).

*DNA methylation in* Lep *and* Ppar-α *promoters in oocytes of obese females*. For *Lep*, the CpG island promoter was hypomethylated in oocytes from CD mice (Figure 2B). The methylation level was significantly higher (*p* < 0.01) in HFD mice compared with CD mice (Figure 2B). For *Ppar-*α, we analyzed 14 CpG sites (Figure 2C) in the CpG island of the *Ppar-*α promoter. At sites 8 and 13, the methylation of *Ppar-α*italic> was significantly lower in HFD mice compared with CD mice (p = 0.13), (Figure 2D). However, at the other sites, DNA methylation levels were obviously lower in HFD mice. The methylation of *Ppar-*α was significantly lower in HFD mice compared with CD mice (*p* < 0.01) (Figure 2D).

**Figure 3:**
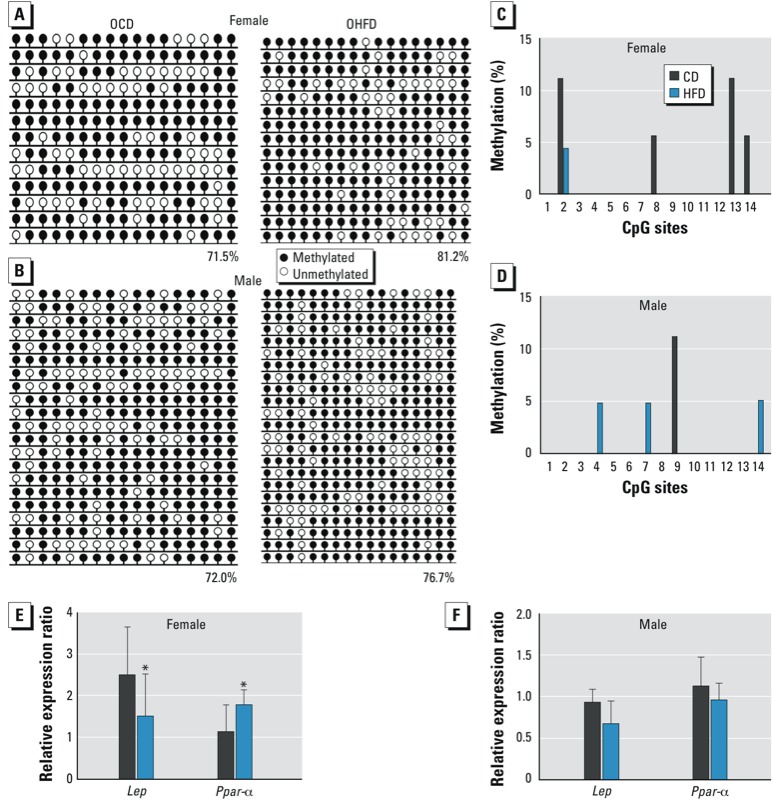
*Lep* and *Ppar-α* methylation status and gene expression in the liver of female and male OCD and OHFD mice at 7–8 weeks of age (*n* = 10 mice from five litters per sex per group). DNA methylation was analyzed by bisulfite sequencing, and gene expression was evaluated by qRT‑PCR. (*A,B*) DNA methylation of Lep in liver of female (*A*) and male (*B*) offspring. Numbers indicate the percentage of methylation; blank loci indicate lost CpG. (*C,D*) DNA methylation at CpG sites of Ppar-α in liver of female (*C*) and male (*D*) offspring. CpG sites are numbered 1–14. (*E,F*) Expression of *Lep* and *Ppar-α* in liver of female (*E*) and male (*F*) offspring. *p < 0.05.

*DNA methylation in* Lep *and* Ppar*-*α *promoters in liver of female offspring.* The methylation level of the *Lep* promoter in female liver was higher in OHFD mice (81.2%) than in OCD mice (71.5%; *p* = 0.013). For male offspring, the methylation level was slightly higher in OHFD mice compared with OCD mice (*p* = 0.138) (Figure 3B). The methylation level at the *Lep* promoter region was similar for females (71.5%) and males (72.0%) in the OCD group (*p* = 0.898). However, in the OHFD group, the methylation level was slightly lower in males than in females (*p* = 0.179).

At CpG sites 2, 8, 13, and 14, the methylation level for *Ppar-*α was decreased in the liver of OHFD females compared with OCD females (Figure 3C). The mean methylation level in the promoter of *Ppar-*α was higher in OCD females than in OHFD females (*p* < 0.05). In livers of male offspring (Figure 3D), the methylation patterns in the *Ppar-*α promoter were similar between the OHFD and the OCD group (*p* = 0.877).

Because the methylation patterns at the CpG island in the promoter region control gene expression for both *Lep* and *Ppar-*α, we further investigated their expressions at the mRNA level. We found that the expression level of *Lep* in the liver of OHFD females was significantly lower than that in OCD females (*p* < 0.05; Figure 3E) but that *Ppar-*α expression was higher in OHFD females than in OCD females (*p* < 0.05, Figure 3E). We found no significant differences in expression of *Lep* and *Ppar-*α in the liver of OHFD and OCD males (*p* = 0.275 and 0.603, respectively; Figure 3F).

**Figure 4:**
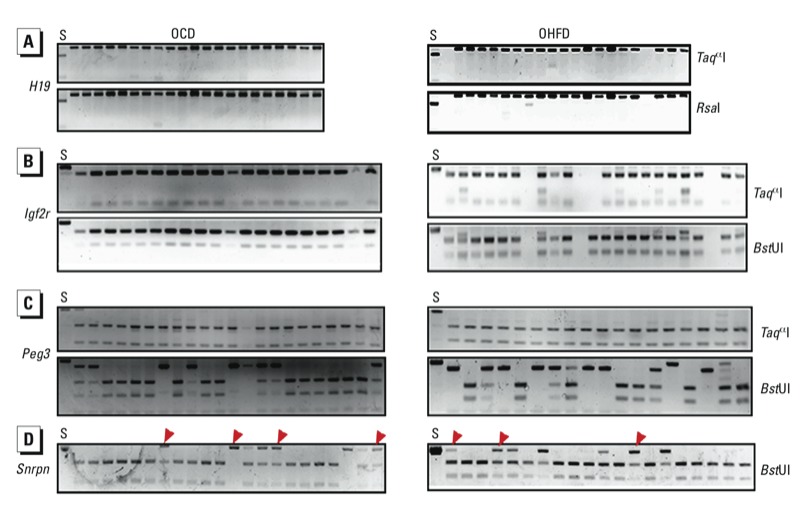
DNA methylation patterns in DMRs of paternally imprinted gene *H19* (*A*) and maternally imprinted genes *Igf2r* (*B*), *Peg3* (*C*), and *Snrpn* (*D*) was in oocytes of OHFD and OCD mice as determined by COBRA. Oocytes from 10 mice were used for each analysis. Spermatozoa (*S*) were used as a control. Restriction enzymes used are shown on the right. Red arrowheads indicate undigested bands.

**Figure 5:**
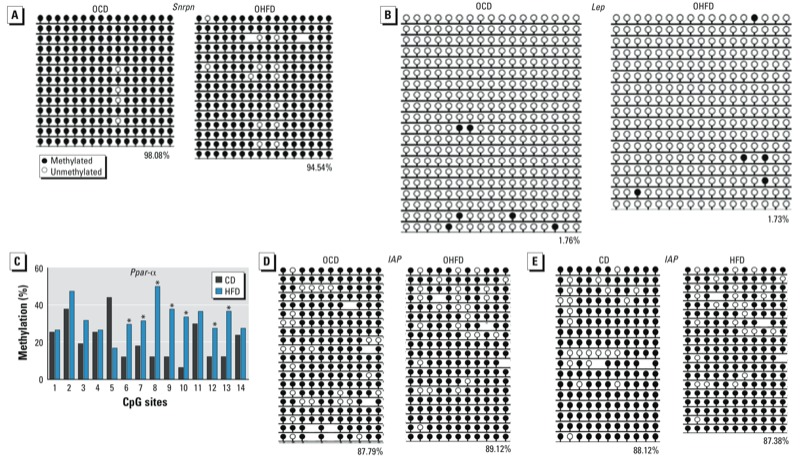
DNA methylation status of *Snrpn*, *Lep*, *IAP* (intracisternal A particle), and *Ppar-α* in oocytes of offspring (*A–D*) and of *IAP* in oocytes of CD and HFD dams (*E*), as analyzed by bisulfite sequencing. Oocytes from 10 mice were used per analysis. (*A–B*) Methylation status of *Snrpn* (*A*) and *Lep* (*B*) in offspring oocytes. (*C*) DNA methylation at CpG sites of *Ppar-α* in offspring oocytes; CpG sites are numbered 1–14. (*D*) Methylation status of *IAP* in offspring oocytes. (*E*) Methylation patterns of *IAP* in oocytes from CD and HFD dams. In (*A,B,D,E*), numbers indicate the percentage of methylation, and blank loci indicate lost CpG. *p < 0.01.

*DNA methylation patterns of imprinted genes in oocytes of offspring.* When we analyzed oocytes for *H19*, *Igf2r*, *Peg3*, and *Snrpn* (approximately 100 oocytes per gene in each group, results showed that their methylation patterns were not altered in oocytes of OHFD females (Figure 4A–C). The differences between the OHFD and OCD groups were not significant (Figure 5A). For *Snrpn* (Figure 4D), some samples were not completely digested by enzymes. Further analysis by bisulfite sequencing showed that this was the result of DNA methylation changes at CpG loci located at the recognition site of *Bst*UI.

*The methylation levels of CpG sites in* Lep *and* Ppar*-*α *promoters in oocytes in offspring*. In our analysis of approximately 100 oocytes per gene in each group, we found that the methylation levels of *Lep* were very low in oocytes from both OCD and OHFD mice (Figure 5B). The methylation levels at CpG sites 6–10, 12, and 13 of the *Ppar-*α promoter were significantly increased in oocytes from OHFD females compared with OCD females (*p* < 0.01; Figure 5C), but methylation levels at other CpG sites were similar.

*The methylation level of IAP elements is not altered in oocytes from obese mothers and their offspring*. We evaluated the methylation status of IAP in oocytes from OCD and OHFD females and their CD and HFD mothers (approximately 100 oocytes/group) by bisulfite sequencing. The methylation level of IAP was similar in the OHFD and OCD groups (Figure 5D) as well as in CD and HFD groups (Figure 5E).

## Discussion

Establishing and maintaining proper DNA methylation is important for normal embryo development and for adult health. Modification of DNA methylation provides a link between the environment and gene expression. Previous studies have shown that malnutrition changed the DNA methylation status (Heijmans et al. 2008; Tobi et al. 2009; Waterland and Jirtle 2003). Our previous studies have revealed that postovulatory aging and maternal diabetes mellitus can alter DNA methylation patterns in DMRs of some imprinted genes in oocytes (Ge et al. 2013; Liang et al. 2008). Overweight and obesity caused mainly by a diet excessively high in fat and a low level of physical activity are among the largest worldwide threats to the health of our population (Finucane et al. 2011). Oocyte quality is decreased in obese mothers, and their children are predisposed to health problems (Igosheva et al. 2010; Jungheim et al. 2010; Minge et al. 2008). In the present study, we found that obesity did not significantly affect DNA methylation in DMRs of selected imprinted genes in oocytes, but that it did alter the DNA methylation levels of the promoters of *Lep* and *Ppar-*α in oocytes.

Offspring of obese mothers are more likely to become obese adults (Howie et al. 2009; Jungheim et al. 2010). Generally, there is no genetic mutation associated with this condition. Obesity is a metabolic disease, and many studies have demonstrated that it is related to epigenetic changes (reviewed by Youngson and Morris 2013). Therefore, in the present study we investigated the DNA methylation levels of *Lep* and *Ppar-*α, which are involved in metabolic processes and are regulated by DNA methylation (Cordero et al. 2011a; Lillycrop et al. 2008). We found that the methylation level in the *Lep* promoter was significantly increased in oocytes of the HFD dams compared with CD dams. The level of *Lep* methylation in the liver was increased in female OHFD mice compared with OCD females. This suggests that the abnormal DNA methylation status in the *Lep* promoter in oocytes from HFD dams may be maintained in the liver of their female offspring. DNA methylation in the promoter controls the expression of *Lep*; if the expression level of *Lep* is lower, the individual tends to gain body weight (Allard et al. 2013; Cordero et al. 2011a). In the present study, we indeed found that *Lep* expression in the liver of OHFD females, corresponding to its higher methylation, was significantly lower than that in OCD females. We found only a slight decrease for male OHFD mice (*p* = 0.275). This is consistent with the average body weight of offspring: Offspring from obese mothers tend to have higher body weight than do offspring from non-obese mothers.

*Ppar-*α is a key factor for controlling systemic energy homeostasis, including adipocyte differentiation, inflammation, energy homeostasis, and lipoprotein and glucose metabolism (Bensinger and Tontonoz 2008; Rakhshandehroo et al. 2010; Stienstra et al. 2007). In the present study, the mean methylation level in the *Ppar-*α promoter was decreased in oocytes of obese dams; in the liver of female OHFD mice, the methylation level of the *Ppar-*α promoter was still lower than in OCD mice, especially at CpG sites 2 and 14. Correspondingly, the expression of *Ppar-*α in the liver of female OHFD mice was obviously higher than in female OCD mice (*p* < 0.05). Although we did not test the metabolism of glucose or lipid, offspring of obese mothers have lower glucose tolerance (Caluwaerts et al. 2007; Magliano et al. 2013). Zhang et al. (2005, 2009) reported that the expression of *Ppar-*α mRNA and protein in liver of OHFD females was higher than that in controls. This is in agreement with our result on the expression of *Ppar-*α in liver. Zhang et al. (2005) reported that triglyceride levels were negatively correlated with the level of *Ppar-*α protein in liver of offspring of obese mothers, and Park and Mun (2013) reported that mice fed a high-fat-diet have a higher *Ppar-*α level and reduced glucose tolerance in liver compared with the controls. This is not contrary to the function of *Ppar-*α because many factors, such as CD36 and CPT-1 (Sato et al. 2002), participate in the process of regulating *Ppar-*α expression and glucose metabolism in the liver (Fischer et al. 2003).

In the present study, the level of DNA methylation in the *Ppar-*α promoter was higher in oocytes of CD females than in the liver of OCD females. We observed a similar pattern in some CpG sites in the *Ppar-*α promoter of oocytes from HFD dams and in the liver of OHFD females. These results indicate that there may be a demethylation process in the *Ppar-*α promoter during embryo development. These findings indicate that changes in DNA methylation may play a key role in overweight and/or obesity of offspring in the mouse model of HFD-induced obesity.

In animal models and in humans, obesity of mothers has been reported to have deleterious influences on the next generation (Dunn and Bale 2009; Gale et al. 2007). To demonstrate how obese mothers transmit the adverse effects to their offspring, we investigated DNA imprinting in oocytes of OHFD mice. We found that DNA methylation patterns in DMRs of *H19, Peg3, Snrpn,* and *Igf2r* in oocytes were similar between OHFD and OCD groups. For *Lep* and IAP, the methylation level was also similar in OHFD oocytes. However, DNA methylation at CpG sites in the *Ppar-*α promoter of oocytes was increased in OHFD females compared with OCD females (Figure 5C).

The methylation status of *Ppar-*α was decreased in oocytes of obese female mice and in the liver of their female offspring, especially at CpG sites 2 and 14. This difference in methylation level in the promoter region of *Ppar-*α between oocytes from HFD females, livers from OHFD females, and oocytes from OHFD females may be induced by the HFD during oocyte maturation and embryo development. During these processes, DNA remethylation and demethylation are prone to being disturbed, and these changes can be inherited by offspring (Bergman and Cedar 2013; Seisenberger et al. 2013; Vrachnis et al. 2012). An adverse uterine environment or deleterious effects of the milk may also be reasons for the differences between offspring of obese mothers and those of nonobese mothers. However, the detailed mechanism is still unknown, and it is not clear whether this change could have effects on future generations.

Several studies have reported that expression of individual gene products is different in the human placenta for male and female fetuses (Lehavi et al. 2005; Steier et al. 2004). Other studies have reported that, in mice fed a low-fat diet or a very-high-fat diet, the female placenta displays more striking changes in gene expression than the male placenta at embryonic days 12.5 and E15.5 (Gallou-Kabani et al. 2010; Mao et al. 2010). In the present study, we found that the level of DNA methylation in *Lep* and *Ppar-*α promoters was significantly altered in the liver of OHFD females, but not in OHFD males, compared with corresponding OCD mice. Gene expression coincided with the methylation pattern. Compared with OCD mice, the mean body weight of OHFD mice at 12 weeks of age increased by 19.4 ± 5.5% for males and 26.3 ± 8.4% for females (*p* = 0.084). The results of sexual dimorphism are consistent with previous findings (Lehavi et al. 2005; Steier et al. 2004). Many factors may play a role in this difference, such as blood flow from the maternal peripheral circulation to the uteroplacental circulation, microRNAs, hormones, growth factors, placental structure and functions, and others (Clifton 2010). However, the detailed mechanism responsible for the differences found in the present study is still obscure.

## Conclusions

We observed that DNA methylation is altered in oocytes of obese (HFD) dams and in the oocytes (female) and livers (female and male) of their offspring. These alterations may partly explain the adverse effects of maternal obesity on reproduction and offspring health.

## Supplemental Material

(217 KB) PDF
