# Erratum: “DNA Methylation in Oocytes and Liver of Female Mice and Their Offspring: Effects of High-Fat-Diet–Induced Obesity”

**DOI:** 10.1289/ehp.122-A150

**Published:** 2014-06-01

Ge et al. noticed an error in the “Results” of their article “DNA Methylation in Oocytes and Liver of Female Mice and Their Offspring: Effects of High-Fat-Diet–Induced Obesity” [Environ Health Perspect 122:159–164 (2014); http://dx.doi.org/10.1289/ehp.1307047]. The sentence “At sites 8 and 13, the methylation of *Ppar*-α was significantly lower in HFD mice compared with CD mice (*p* < 0.01) (Figure 2D)” was incorrect. The corrected sentence is as follows: “The methylation of *Ppar*-α was significantly lower in HFD mice compared with CD mice (*p* < 0.01) (Figure 2D).”

*EHP* regrets the error.

