# Supplementary material for: DNA Methylation in Oocytes and Liver of Female Mice and Their Offspring: Effects of High-Fat-Diet–Induced Obesity
Source: Environ Health Perspect. 2013 Dec 6;122(2):159–64. doi: 10.1289/ehp.1307047 (PMC3915265; doi:10.1289/ehp.1307047)
Supplement: (217 KB) PDF [file ehp.1307047.s001.pdf]

Environmental Health Perspectives doi:10.1289/ehp.1307047

Supplemental Material

DNA Methylation in Oocytes and Liver of Female Mice and Their Offspring: Effects of High-Fat-Diet–Induced Obesity

Zhao-Jia Ge, Shi-Ming Luo, Fei Lin, Qiu-Xia Liang, Lin Huang, Yan-Chang Wei, Yi Hou,

Zhi-Ming Han, Heide Schatten, and Qing-Yuan Sun

Table of Contents

Supplemental Material, Figure 1……………………………………………………………..….2
Supplemental Material, Table 1…………………………………………………………………3
Supplemental Material, Table 2.…………………………………………………………………4
Reference…………………………………………………………………………………………5

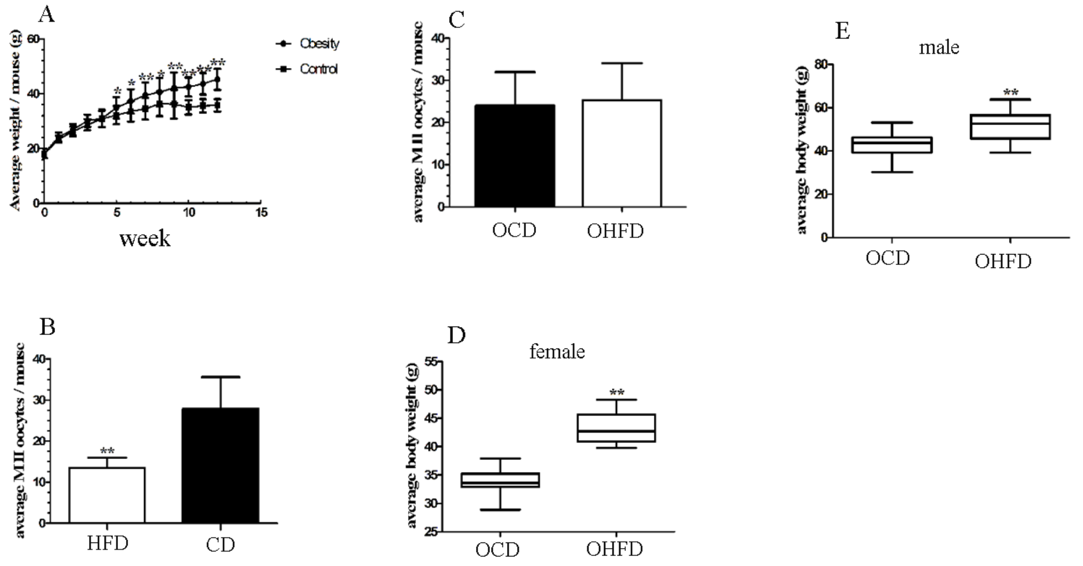

Supplemental Material, Figure S1. Average body weight and average number of MII oocytes per mouse. (A) Average body weight per female mouse fed HFD or CD diet was measured each week and represented as mean ± SD; (B) mice of HFD (n = 81) and CD (n = 83) were super-ovulated and MII oocytes were collected. Average number of MII oocytes per mouse was counted and represented as mean ± SD. White bar, HFD; black bar, CD. (C) The number of MII oocytes per mouse in OHFD (n = 72) and OCD (n = 70) groups was evaluated and represented as mean ± SD; White bar, OHFD; black bar, OCD. (D, E) Average body weight of 12 week-old offspring for female (n = 48) and male (n = 47) was evaluated and represented as mean ± SD. *P < 0.05, **P < 0.01.

Supplemental Material, Table S1. Composition of D12492 and control diet (CD).

Component	D12492	CD
**Macronutrient (%)**		
Protein	26.2	27
Carbohydrate	26.3	48
Fat	34.9	4
**Ingredient (gm)**		
Casein	200	200
L-Cystine	3	3
Corn Starch	0	325
Maltodextrin	125	36
Sucrose	68.8	350
Cellulose	50	50
Soybean Oil	25	23
Lard	245	20
Mineral Mix	10	10
DiCalcium Phosphate	13	12
Calcium Carbonate	5.5	5.5
Potassium Citrate	16.5	16
Vitamin Mix	10	9.8
Choline Bitartrate	2	1.7
Total	773.8	1062

Note: gm, gram.

Supplemental Material, Table S2. Oligonucleotides utilized for PCR.

Genes	Primer name	Primer sequence
H19	Forward1	5′-GAGTATTTAGGAGGTATAAGAATT-3′
H19	Reverse1	5′-ATCAAAAACTAACATAAACCCCT-3′
H19	Forward2	5′-GTAAGGAGATTATGTTTATTTTTGG-3′
H19	Reverse2	5′-CCTCATTAATCCCATAACTAT-3′
Peg1	Forward1	5′-GATTTGTTATATAAAGGTTAATGAG-3′
Peg1	Reverse1	5′-TCATTAAAAACACAAACCTCCTTTAC-3′
Peg1	Forward2	5′-TTTTAGATTTTGAGGGTTTTAGGTTG-3′
Peg1	Reverse2	5′-AATCCCTTAAAAATCATCTTTCACAC-3′
Peg3	Forward1	5′-TGATAATAGTAGTTTGATTGGTAGGG-3′
Peg3	Reverse1	5′-TAATTCACACCTAAACCCTAAAACC-3′
Peg3	Forward2	5′-TTTTGTAGAGGATTTTGATAAGGAGG-3′
Peg3	Reverse2	5′-AAATACCACTTTAAATCCCTATCACC-3′
Snrpn	Forward1	5′-TATGTAATATGATATAGTTTAGAAATTAG-3′
Snrpn	Reverse1	5′-AATAAACCCAAATCTAAAATATTTTAATC-3′
Snrpn	Forward2	5′-AATTTGTGTGATGTTTGTAATTATTTGG-3′
Snrpn	Reverse2	5′-ATAAAATACACTTTCACTACTAAAATCC-3′
Igf2r	Forward1	5′-TTA GTGGGGTATTTTTATTTGTATGG-3′
Igf2r	Reverse	5′-AAATATCCTAAAAATACAAACTACAC-3′
Igf2r	Forward2	5′-GTGTGGTATTTTTATGTATAGTTAGG-3′
Leptin	Forward1	5′-TGTAAAGAGTTGTCGGAAAAAGTAG-3′
Leptin	Reverse1	5′-CTATCCACATAACCTCCTTCTTACCTCA-3′
Leptin	Forward2	5′-TTGGAAGTATTATTTTAAGGGATT-3′
Leptin	Reverse2	5′-TTATAACTACCCCAATACCACTTAC-3′
Ppar-α	Forward1	5′-GGTATTTTGGTTATTTGTTG-3′
Ppar-α	Reverse1	5′-AACCCCTAAACACCTAAAACTA-3′
Ppar-α	Forward2	5′-TTTTGGGATTTTAAAGATTAGATTT-3′
Ppar-α	Reverse2	5′-CTAAACACCTAAAACTACACTC-3′
IAP	Forward1	5′-TTGATAGTTGTGTTTTAAGTGGTAAATAAA-3′
IAP	Reverse1	5′-AAAACACCACAAACCAAAATCTTCTAC-3′
IAP	Forward2	5′-TTGTGTTTTAAGTGGTAAATAAATAATTTG-3′
IAP	Reverse2	5′-CAAAAAAAACACCACAAACCAAAAT-3′
GADPH	Forward	5′-TGACCTCAACTACATGGTCTACA-3′
GADPH	Reverse	5′-CTTCCCATTCTCGGCCTTG-3′
Leptin	Forward	5′-GTGGCTTTGGTCCTATCTGTC-3′
Leptin	Reverse	5′-CGTGTGTGAAATGTCATTGATCC-3′
Ppar-α	Forward	5′-AACATCGAGTGTCGAATATGTGG-3′
Ppar-α	Reverse	5′-CCGAATAGTTCGCCGAAAGAA-3′

Reference

Ge ZJ, Liang XW, Guo L, Liang QX, Luo SM, Wang YP, et al. 2013. Maternal diabetes causes alterations of DNA methylation statuses of some imprinted genes in murine oocytes. Biol Reprod 88:117.
